# Preliminary clinical and radiological evaluation of osteosynthesis using the Femoral Neck System (FNS) for subcapital fractures of the femur

**DOI:** 10.1038/s41598-024-64955-z

**Published:** 2024-06-24

**Authors:** Jose Manuel Hernández-Naranjo, Borja Campuzano-Bitterling, Marina Renau-Cerrillo, Marian Vives-Barquiel, María Pilar Camacho-Carrasco, Ernesto Muñoz-Mahamud

**Affiliations:** Department of Orthopedics and Trauma Surgery, Hospital Clinic of Barcelona, University of Barcelona, Barcelona, Spain

**Keywords:** Medical research, Outcomes research

## Abstract

Addressing subcapital fractures of the femur poses a substantial clinical challenge, complicated by the diverse range of available osteosynthesis materials. This study is dedicated to a comprehensive analysis of the clinical and radiological implications linked with the implementation of the Femoral Neck System (FNS) in osteosynthesis procedures. A descriptive study was conducted involving patients who underwent osteosynthesis for subcapital fractures of the femur utilizing the FNS during the period from 2019 to 2022. The investigation encompassed various facets, including the classification of fractures according to the Garden and Pauwells classifications, criteria for achieving precise reduction based on the Garden criteria and Tip Apex Distance (TAD). At the one-year follow-up, factors such as fracture consolidation, loss of reduction, fracture collapse, complications, and functional outcomes were evaluated utilizing the Harris Hip Score (HHS) scale. The study cohort included a total of 26 patients, among whom 22 exhibited non-displaced subcapital femur fractures categorized as Garden I and II. Successful reduction was accomplished in 23 cases, in which 24 cases (92.3%) displayed a TAD measurement below 25 mm. According to the HHS, patients achieved an average score of 90.9 (ranging from 63 to 100) following the surgical intervention, with predominantly "excellent" and "good" outcomes. The outcomes derived from our investigation corroborate the viability of the Femoral Neck System (FNS) as a reliable option for osteosynthesis in femoral neck fractures. The results obtained are comparable to those achieved with other available implants, as highlighted by previous studies.

## Introduction

Subcapital femur fractures stand as a prevailing concern within the domains of orthopedic surgery and traumatology, presenting a progressively increasing incidence^1^. These fractures lend themselves to epidemiological categorization, revealing two distinctive cohorts based on age and bone quality. The first group encompasses young patients with good bone quality and a history of high-energy traumas, such as vehicular accidents or falls. In contrast, the second group comprises elderly individuals marked by compromised bone quality and a predilection for low-energy traumas.

For patients below the age of 65, a consensus within treatment guidelines advocates for the pursuit of osteosynthesis as a means to preserve the native hip^2^. This therapeutic approach, while preserving the anatomical integrity of the hip joint, incurs the inherent risk of potential complications such as non-union or avascular necrosis of the femoral head, scenarios that could potentially necessitate subsequent salvage procedures involving total hip arthroplasty. Mitigating these complications hinges upon the attainment of anatomical reduction during the surgical intervention and the judicious application of synthesis techniques.

In the context of elderly patients, a degree of uncertainty prevails regarding the appropriateness of osteosynthesis, notwithstanding the augmented vulnerability to complications and reoperation due to diminished bone quality. However, among the elderly demographic, arthroplasty (comprising hemiarthroplasty and total hip arthroplasty), emerges as the preferred modality of treatment. However, the execution of arthroplasty in the setting of hip fractures carries elevated risks compared to elective scenarios^3^.

Within the realm of osteosynthesis, a spectrum of therapeutic modalities is available. A prominent strategy involves the utilization of three cannulated screws configured in an inverted triangle pattern, typically anchored from the lateral cortex of the femur. This approach, however, deviates in cases of Pauwells Type III fractures and select Type II fractures, where the adoption of a sliding hip screw and a cannulated screw is favored. This alteration aims to enhance control over shear forces that could jeopardize the efficacy of fixation using cannulated screws^4^. Patients subjected to osteosynthesis often confront a heightened incidence of complications, prominently encompassing avascular necrosis of the femoral head and non-union, particularly when the attainment of precise reduction proves intricate^5^. The presence of pre-existing hip osteoarthritis shifts the therapeutic consideration toward the inclination for hip arthroplasty over osteosynthesis^6,7^. The decision-making process is further nuanced by the patient's level of dependency and concurrent comorbidities, which collectively contribute to shaping the optimal therapeutic trajectory.

A new osteosynthesis system, the Femoral Neck System (FNS, DePuy Synthes, West Chester, PA, USA), has been introduced for subcapital femur fractures. It preserves fracture reduction achieved during surgery due to its advanced design, offering better control over forces. This system works for various subcapital femur fractures regardless of Pauwells classification. It also has benefits like minimally invasive approach, smaller bone footprint, controlled fracture collapse, and prevention of implant protrusion in collapse cases.

Additional research is required to establish if this enhanced fracture stability results in a reduced occurrence of issues like non-union and avascular necrosis of the femoral head. The aim of this retrospective study is to evaluate both the safety of the implant and the complications in the medium term.

## Material and methods

A descriptive retrospective observational study was conducted, including patients diagnosed with subcapital fractures of the femur surgically treated with FNS between 2019 and 2022, with the following inclusion criteria: age over 18 years, minimum follow-up of 12 months and patients able to walk before sustaining the fracture. The initial fracture diagnosis was made through simple anteroposterior and axial radiographs of the affected hip. Clinical and sociodemographic characteristics of the patients were collected, including sex, age, ASA (American Society of Anesthesiologists) risk, mechanism of injury, laterality, blood test data at admission and 24 h after surgery, and fracture type according to the Garden and Pauwels classifications. Prophylaxis for thromboembolic disease was administered with 40 mg of subcutaneous low molecular weight heparin every 24 h. Infection prophylaxis was performed with a single dose of 1500 mg cefuroxime and 800 mg teicoplanin, following the implant surgery protocol, 30 min before anesthesia induction.

Patients were operated on by experienced fracture surgeons. The surgical technique was the same in all cases, following a standardized protocol: the patient was placed on an orthopedic traction table, closed reduction maneuvers were performed to achieve reduction according to Garden's criteria, a direct lateral approach was made to the proximal femur region, a 3.2 mm diameter cervico-cephalic Kirschner wire (KW) was placed with a 130º guide supported on the lateral cortex, seeking a center-center position in anteroposterior and axial radiographic projections, an anti-rotational KW was placed through the fracture, the length of the cervico-cephalic bolt was measured to maintain a correct Tip Apex distance, the KW was drilled through the lateral cortex of the femur and femoral neck until the predetermined measurement, the FNS implant (Synthes^®^, Switzerland) of the corresponding size was inserted and aligned with the femoral diaphysis, the anti-rotational screw was drilled, the anti-rotational screw was inserted, the anti-rotational KW was removed, the screw in the plate was drilled and measured, and fixation was performed with a 5.0 mm diameter locking screw. If necessary, compression of the fracture was achieved through the anti-rotational screw. Sitting was allowed at 24 h post-surgery, and partial weight-bearing ambulation with crutches or a walker was permitted based on tolerance of the patient. Full weight-bearing was allowed between 4 and 6 weeks at the surgeon's discretion.

During the hospital stay, hemoglobin (Hb) and hematocrit levels were analyzed to assess blood loss, and the need for transfusion and complications were recorded. Clinical and radiological follow-up of the patients was conducted at 1, 3, 6, and 12 months after surgery, with the minimum follow-up being 12 months. The final functional outcomes were assessed using the HHS, and simple radiographs were used to evaluate the maintenance of fracture alignment, fracture collapse, signs of femoral head necrosis, and fracture consolidation.

## Results

A total of 26 patients were included in the study. Main characteristics are depicted in Table 1. Out of the 26 patients, 14 were male (53.8%) and the mean age was 60.1 years (range: 30–89 years). Regarding the fracture mechanism, 13 (50%) occurred due to a casual fall from the patient's own height, while 11 (42.3%) were caused by various high-energy mechanisms. Of the total 26 fractures, 14 occurred on the right side (53.8%), and 12 (46.2%) on the left side. According to the Garden Classification, 4 fractures were displaced (Garden III and IV, totaling 15.4%), and the other 22 (84.6%) were non-displaced (Garden I and II fractures). Concerning the Pauwels classification, 4 were Type I (15.4%), 14 were Type II (53.8%), and 8 (30.8%) were Type III.

**Table 1:** Clinical and radiological characteristics of the patients included in the study.

	Sex	Age	Side	Garden	Pauwels	Mechanism	Energy	TAD	Collapse in mm	Previous HHS	Final HHS	HHS rating	Complications
*1*	F	51	Right	3	3	Hit by a car	High	< 25 mm	8	100	96	Excellent	
*2*	M	51	Right	2	3	Ground-level fall	Low	< 25 mm	1	100	100	Excellent	
*3*	M	42	Right	1	3	Skiing	High	> 25 mm	0	100	96	Excellent	
4	M	30	Right	3	3	Skating	High	< 25 mm	1	100	96	Excellent	
*5*	M	68	Left	2	2	Bicycle	High	< 25 mm	8	93	93	Excellent	
*6*	F	77	Right	2	1	Fall down the stairs	High	< 25 mm	14	98	97	Excellent	
*7*	F	51	Right	2	2	Fall on the bus	High	< 25 mm	2	100	92	Excellent	
*8*	M	81	Right	1	1	Ground-level fall	Low	< 25 mm	5	69	65	Poor	Passed away after 6 months
*9*	M	39	Left	1	2	Skating	High	< 25 mm	1,5	100	96	Excellent	
*10*	F	59	Right	1	1	Ground-level fall	Low	< 25 mm	2	86	63	Poor	Avascular necrosis
*11*	F	84	Right	2	2	Ground-level fall	Low	> 25 mm	0	89	85	Good	
*12*	M	29	Right	2	2	Falling down embankment	High	< 25 mm	1	100	100	Excellent	
*13*	M	48	Right	1	2	Bicycle	High	< 25 mm	0	100	96	Excellent	
*14*	M	54	Left	2	3	Bicycle	High	< 25 mm	1	100	100	Excellent	
*15*	F	73	Left	2	1	Ground-level fall	Low	< 25 mm	0	100	93	Excellent	
*16*	F	79	Left	1	2	Ground-level fall	Low	< 25 mm	1	100	78	Fair	
*17*	F	88	Left	1	2	Ground-level fall	Low	< 25 mm	3	100	80,2	Good	
*18*	M	43	Left	1	2	Ground-level fall	Low	< 25 mm	2	100	96	Excellent	
*19*	F	56	Left	1	2	Ground-level fall	Low	< 25 mm	0	100	90	Excellent	
*20*	F	89	Left	1	2	Syncope	Low	< 25 mm	0	91	77	Fair	
*21*	M	58	Right	1	2	Ground-level fall	Low	< 25 mm	2	100	95	Excellent	
*22*	F	48	Right	4	3	Stress fracture	Low	< 25 mm	8	100	100	Excellent	
*23*	F	64	Left	1	2	Ground-level fall	Low	< 25 mm	0	100	90,6	Excellent	
*24*	M	63	Right	1	2	Ground-level fall	Low	< 25 mm	0	100	100	Excellent	
*25*	F	76	Left	2	3	Ground-level fall	Low	< 25 mm	18	100	94	Excellent	Non-union
*26*	M	43	Left	4	3	Skating	High	< 25 mm	10	100	94	Excellent	Non-union

*F* female, *M* male.

**Figure 1:**
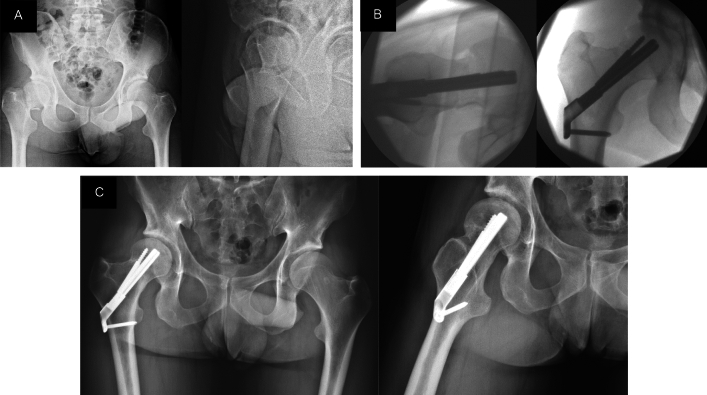
(**A**) AP and axial radiographs of the pelvis and right hip were taken in the emergency department of a 29-year-old patient who suffered a Garden III subcapital fracture and Pauwels III fracture of the right femur. (**B**) Intraoperative fluoroscopy controls with AP and axial views. Correct reduction of the fracture was observed in both the AP and axial projections. (**C**) AP and axial hip radiographs during the annual follow-up in the clinic. Complete consolidation of the fracture was observed.

Based on Garden's reduction criteria, correct reduction was achieved in 23 patients (88.5%). The Tip Apex Distance (TAD) was < 25 mm in 24 cases (92.3%). Mean collapse measured in the radiographs at the end of the follow-up period was 3.6 mm (ranging from 0 to 18 mm). Femoral neck collapse occurred in 18 patients (69.2%), and 6 out of the 18 (33.3%) experienced a collapse greater than 5 mm (Fig. 1).

Out of all the patients included in the study, one patient passed away after 6 months from the surgery.

Regarding the functional outcomes measured using the HHS, patients obtained an average score of 90.9 (range between 63 and 100) after surgery. The results were "excellent" in 20 out of 26 patients (76.9%). Among the remaining 6 patients, 2 cases were rated as "good" (7.7%), 2 as "fair" (7.7%), and 2 as "poor" (7.7%). However, only 1 patient showed a decrease in functional category from "good" to "poor" after surgery, while the rest maintained their pre-surgery functional category. The overall mean decrease in the HHS for all patients, comparing the score before and after surgery, was 6.3 (range: 0–23).

**Figure 2:**
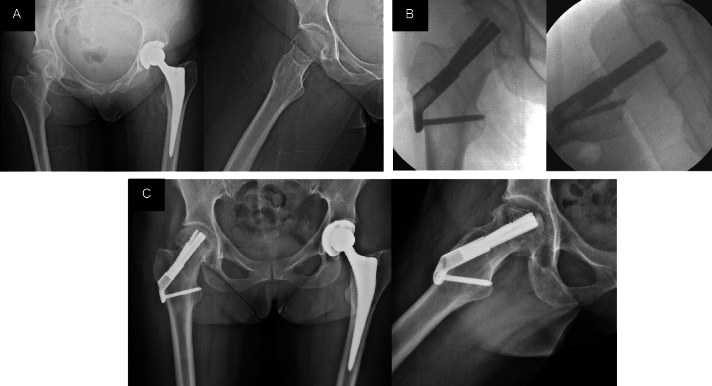
(**A**) AP and axial hip radiographs taken in the emergency department. (**B**) Intraoperative fluoroscopy controls with AP and axial views. (**C**) AP and axial hip radiographs at the 6-month follow-up. Articular surface collapse and protrusion of the bolt and anti-rotational screw are observed.

The aforementioned patient with functional deterioration involved a 59-year-old female patient who had sustained a right subcapital femur fracture classified as Garden I, with a Pauwels classification of 1, resulting from an innocuous fall. Her medical history was notably marked by a left total hip replacement undertaken six years prior, a response to avascular necrosis afflicting the left femoral head. Although meticulous reduction was achieved during the surgical intervention for the subcapital fracture of the femur, follow-up radiographs after discharge unveiled signs of femoral head necrosis. Concomitantly, the patient presented pain and an impaired gait, thus the patient underwent revision surgery, entailing the removal of the FNS and the subsequent hip arthroplasty procedure (Fig. 2).

Additionally, there were two other cases in which follow-up radiographs revealed varus collapse and non-union. These patients experienced progressive pain and difficulty in walking, leading to the removal of the FNS system and subsequent hip arthroplasty. After the arthroplasty, both cases showed excellent functional outcomes according to the Harris Hip Score (94 points). Overall, there were 3 cases of complications (11.5%).

Informed consent was waived due to retrospective nature of the study. This study was approved by the Drug Research Ethics Committee of our center under the code HCB/2022/0080 and it does not contain any studies with human participants or animals performed by any of the authors. All methods were carried out in accordance with relevant guidelines and regulations.

## Discussion

Within this study, we present the outcomes derived from 26 patients undergoing treatment for femoral neck fractures, using the FNS within the period from 2019 to 2022. While it is acknowledged that the size of our patient cohort may not be extensive, the discernible clinical value is derived from the robust confirmation of the FNS system's safety and efficacy, effectively underpinned by substantive clinical and radiological data.

Furthermore, in our study there are a total of 9 patients over 65 years old; however, all of them had non-displaced femoral neck fractures (Garden I and Garden II), and in this specific group of patients the optimal treatment is still a subject of debate^8,9^. According to the literature, in the presence of certain specific risk factors such as femoral neck comminution, corticosteroids, degenerative changes in the hip, and others (alcohol dependence, osteoporosis, rheumatoid arthritis, chronic kidney disease), there is a higher risk of osteosynthesis failure, so arthroplasty would be the treatment of choice^10^. However, this is not the case for the patients in our study and of the 9 patients mentioned only 1 required total hip arthroplasty due to avascular necrosis of the femoral head.

The FNS tackles the challenge of combining the advantages of the two main options currently available for osteosynthesis in femoral neck fractures. On one hand, it aims to be minimally invasive, similar to cannulated screws, while on the other hand, it strives to achieve greater stability, similar to the DHS (dynamic hip screw) system. The design of the FNS implant allows for surgical placement with a small incision, minimizing the bone footprint. By incorporating a plate with a locking screw and a cephalic bolt, it provides angular stability and resistance against shear forces, which is the main weakness of cannulated screws. From the same point of entry of the cephalic bolt, a divergent anti-rotational screw provides rotational stability, even in cases of small femoral necks. Both the anti-rotational screw and the cephalic bolt feature a dynamic design, allowing controlled collapse of the fracture up to 20 mm without lateral protrusion of the implant, reducing the risk of limb length discrepancy and deterioration of functional outcomes after fracture consolidation.

Stoffel et al.^11^ compared the biomechanical properties of the FNS with the DHS system and cannulated screws in the treatment of Pauwels III femoral neck fractures. Their findings underscored the biomechanical superiority of both the FNS and DHS systems when juxtaposed with cannulated screws, a distinction holding statistical significance. Notably, in the comparison between the FNS and DHS systems, no statistically meaningful disparities in biomechanical performance were detected.

Femoral neck shortening, consequent to fracture impaction, can precipitate the weakening of abductor muscles, driven by limb shortening and the ensuing diminution of hip offset^12^. This can precipitate manifestations such as discomfort, muscular debilitation, and patient dissatisfaction. In our patients, collapse occurred in 18 patients (69.2%), and 6 out of 16 had a collapse greater than 5 mm (33.3%). Although some studies conclude that greater femoral neck shortening is associated with worse functional outcomes, in our study all 6 mentioned patients had "excellent" functional results according to the HHS. The only case of functional score deterioration in the HHS occurred in the patient with avascular necrosis of the femoral head and non-union of the fracture.

The rate of reintervention in patients with femoral neck fractures varies in the literature, ranging from 8 to 42%^13,14^. In our study, among the 26 patients treated using the FNS system, only 3 of them required reintervention (11.5% of the total), placing our reintervention rate at the lower end of those reported in the literature.

In the FAITH study, a randomized controlled trial of 1108 patients with femoral neck fractures treated with the DHS or cannulated screws, femoral head necrosis was more frequent in patients treated with the DHS system compared to those treated with cannulated screws^15^. This could be explained by the larger diameter of the DHS system compared to cannulated screws. However, it could also be a bias, as the DHS system is usually used in more unstable fractures. The FNS system consists of a central 10 mm diameter bolt and a 6.4 mm anti-rotational screw. Future studies should assess how the FNS system affects the vascularization of the femoral head.

Tang et al. compared the results of the FNS system with cannulated screws in the treatment of femoral neck fractures, obtaining similar complication rates (non-union, avascular necrosis, and loosening) between the two groups^16^. Moreover, in some studies, the FNS system has shown a lower complication rate^17–19^. Subsequently, Rajnish et al. conducted a meta-analysis that included a total of 6 retrospective studies with 371 patients, comparing the outcomes of cannulated screws versus the FNS system in the treatment of femoral neck fractures. They concluded that the rate of complications such as implant failure, non-unions, and avascular necrosis was similar in both groups^20^.

The major drawback of the current study is related to its retrospective nature, so the results feature several inherent limitations. The second limitation is that different surgeons participated in the study and there may be significant differences in their intra-operative management. However, all the surgeons used the same surgical procedure and the same surgical technique. Finally, the limited number of cases as a consequence of a single center study addressing a low prevalent pathology; require future studies to corroborate our findings.

We conclude that the FNS is a safe and option for the treatment of femoral neck fractures, regardless of the degree of displacement and fracture line verticality. Although the superiority of this internal fixation system cannot be accepted unless there is a large sample size, long-term follow-up, and comparative studies with other devices, the results obtained are comparable to those achieved with other available implants, as highlighted by previous studies in the literature.

## Data Availability

The datasets used and analysed during the current study are available from the corresponding author on reasonable request.

## References

[1] Sheehan SE, Shyu JY, Weaver MJ, Sodickson AD, Khurana B (2015). Proximal femoral fractures: What the orthopedic surgeon wants to know. Radiographics.

[2] Florschutz AV, Langford JR, Haidukewych GJ, Koval KJ (2015). Femoral neck fractures: Current management. J. Orthop. Trauma.

[3] Parvizi J, Ereth MH, Lewallen DG (2004). Thirty-day mortality following hip arthroplasty for acute fracture. J. Bone Jt. Surg. Am..

[4] Lim EJ, Shon H-C, Cho J-W, Oh J-K, Kim J, Kim C-H (2021). Dynamic hip screw versus cannulated cancellous screw in Pauwels type II or type III femoral neck fracture: A systematic review and meta-analysis. J. Pers. Med..

[5] Krischak G, Beck A, Wachter N, Jakob R, Kinzl L, Suger G (2003). Relevance of primary reduction for the clinical outcome of femoral neck fractures treated with cancellous screws. Arch. Orthop. Trauma Surg..

[6] Schmidt AH, Leighton R, Parvizi J, Sems A, Berry DJ (2009). Optimal arthroplasty for femoral neck fractures: Is total hip arthroplasty the answer?. J. Orthop. Trauma.

[7] Miller BJ, Callaghan JJ, Cram P, Karam M, Marsh JL, Noiseux NO (2014). Changing trends in the treatment of femoral neck fractures: A review of the american board of orthopaedic surgery database. J. Bone Jt. Surg. Am..

[8] Okike K, Udogwu UN, Isaac M, Sprague S, Swiontkowski MF, Bhandari M, Slobogean GP, on behalf of the FAITH Investigators (2019). Not all Garden-I and II femoral neck fractures in the elderly should be fixed: Effect of posterior tilt on rates of subsequent arthroplasty. J. Bone Jt. Surg. Am..

[9] Lowe JA, Crist BD, Bhandari M, Ferguson TA (2010). Optimal treatment of femoral neck fractures according to patient’s physiologic age: An evidence-based review. Orthoped. Clin. N. Am..

[10] Melisik M, Hrubina M, Daniel M, Cibula Z, Rovnak M, Necas L (2021). Ultra-short cementless anatomical stem for intracapsular femoral neck fractures in patients younger than 60 years. Acta Orthopaedica Belgica.

[11] Stoffel K, Zderic I, Gras F, Sommer C, Eberli U, Mueller D (2017). Biomechanical evaluation of the femoral neck system in unstable Pauwels III femoral neck fractures: a comparison with the dynamic hip screw and cannulated screws. J. Orthop. Trauma.

[12] Felton J, Slobogean GP, Jackson SS, Della Rocca GJ, Liew S, Haverlag R (2019). Femoral neck shortening after hip fracture fixation is associated with inferior hip function: Results from the FAITH trial. J. Orthop. Trauma.

[13] Rogmark C, Carlsson A, Johnell O, Sernbo I (2003). A prospective randomised trial of internal fixation versus arthroplasty for displaced fractures of the neck of the femur. Functional outcome for 450 patients at two years. J. Bone Jt. Surg..

[14] Oñativia IJ, Slulittel PAI, Diaz Dilernia F, Gonzales Viezcas JM, Vietto V, Ramkumar PN (2018). Outcomes of nondisplaced intracapsular femoral neck fractures with internal screw fixation in elderly patients: A systematic review. Hip Int..

[15] Nauth A, Creek AT, Zellar A, Lawendy A-R, Dowrick A, Gupta A (2017). Fracture fixation in the operative management of hip fractures (FAITH): An international, multicentre, randomised controlled trial. Lancet.

[16] Tang Y, Zhang Z, Wang L, Xiong W, Fang Q, Wang G (2021). Femoral neck system versus inverted cannulated cancellous screw for the treatment of femoral neck fractures in adults: A preliminary comparative study. J. Orthop. Surg. Res..

[17] Zhou X-Q, Li Z-Q, Xu R-J, She Y-S, Zhang X-X, Chen G-X (2021). Comparison of early clinical results for femoral neck system and cannulated screws in the treatment of unstable femoral neck fractures. Orthop. Surg..

[18] Hu H, Cheng J, Feng M, Gao Z, Wu J, Lu S (2021). Clinical outcome of femoral neck system versus cannulated compression screws for fixation of femoral neck fracture in younger patients. J. Orthop. Surg. Res..

[19] Nibe Y, Matsumura T, Takahashi T, Kubo T, Matsumoto Y, Takeshita K (2022). A comparison between the femoral neck system and other implants for elderly patients with femoral neck fracture: A preliminary report of a newly developed implant. J. Orthop. Sci..

[20] Rajnish RK, Srivastava A, Rathod PM, Haq RU, Aggarwal S, Kumar P (2022). Does the femoral neck system provide better outcomes compared to cannulated screws fixation for the management of femoral neck fracture in young adults? A systematic review of literature and meta-analysis. J. Orthop..

